# A comprehensive review of advances in hepatocyte microencapsulation: selecting materials and preserving cell viability

**DOI:** 10.3389/fimmu.2024.1385022

**Published:** 2024-04-17

**Authors:** Hailian Wang, Lebin Wen, Fengdi Jiang, Pengyu Ren, Yixin Yang, Siyuan Song, Zhengteng Yang, Yi Wang

**Affiliations:** ^1^ Clinical Immunology Translational Medicine Key Laboratory of Sichuan Province, Center of Organ Transplantation, Sichuan Academy of Medical Science and Sichuan Provincial People’s Hospital, Chengdu, China; ^2^ Department of Thyroid, Sichuan Second Hospital of Traditional Chinese Medicine, Chengdu, Sichuan, China; ^3^ School of Medicine, University of Electronic Science and Technology of China, Chengdu, China; ^4^ Department of Clinical Medicine, The First Clinical Medical College of Norman Bethune University of Medical Sciences, Jilin, China; ^5^ Department of Neuroscience, Baylor College of Medicine, Houston, TX, United States; ^6^ Department of Pharmacy, Guangxi University of Chinese Medicine, Nanning, China; ^7^ Clinical Immunology Translational Medicine Key Laboratory of Sichuan Province, Sichuan Academy of Medical Sciences and Sichuan Provincial People’s Hospital, School of Medicine, University of Electronic Science and Technology of China, Chengdu, China

**Keywords:** hepatocyte encapsulation, microencapsulation, coculture, xenotransplantation, alginate

## Abstract

Liver failure represents a critical medical condition with a traditionally grim prognosis, where treatment options have been notably limited. Historically, liver transplantation has stood as the sole definitive cure, yet the stark disparity between the limited availability of liver donations and the high demand for such organs has significantly hampered its feasibility. This discrepancy has necessitated the exploration of hepatocyte transplantation as a temporary, supportive intervention. In light of this, our review delves into the burgeoning field of hepatocyte transplantation, with a focus on the latest advancements in maintaining hepatocyte function, co-microencapsulation techniques, xenogeneic hepatocyte transplantation, and the selection of materials for microencapsulation. Our examination of hepatocyte microencapsulation research highlights that, to date, most studies have been conducted *in vitro* or using liver failure mouse models, with a notable paucity of experiments on larger mammals. The functionality of microencapsulated hepatocytes is primarily inferred through indirect measures such as urea and albumin production and the rate of ammonia clearance. Furthermore, research on the mechanisms underlying hepatocyte co-microencapsulation remains limited, and the practicality of xenogeneic hepatocyte transplantation requires further validation. The potential of hepatocyte microencapsulation extends beyond the current scope of application, suggesting a promising horizon for liver failure treatment modalities. Innovations in encapsulation materials and techniques aim to enhance cell viability and function, indicating a need for comprehensive studies that bridge the gap between small-scale laboratory success and clinical applicability. Moreover, the integration of bioengineering and regenerative medicine offers novel pathways to refine hepatocyte transplantation, potentially overcoming the challenges of immune rejection and ensuring the long-term functionality of transplanted cells. In conclusion, while hepatocyte microencapsulation and transplantation herald a new era in liver failure therapy, significant strides must be made to translate these experimental approaches into viable clinical solutions. Future research should aim to expand the experimental models to include larger mammals, thereby providing a clearer understanding of the clinical potential of these therapies. Additionally, a deeper exploration into the mechanisms of cell survival and function within microcapsules, alongside the development of innovative encapsulation materials, will be critical in advancing the field and offering new hope to patients with liver failure.

## 1 Introduction

### 1.1 The evolution of liver transplantation

The liver, one of the human body’s largest and most versatile organs, is incredible for being able to detoxificate, metabolize, and maintain complex interactions with other organs like the kidney and spleen. Despite its critical role, individuals suffering from liver-based metabolic disorders (LBMD), hepatocellular carcinoma, fulminant liver failure, and end-stage liver diseases often face limited treatment options. The landscape of liver disease treatment underwent a significant transformation in 1963 when Thomas E. Starzl and his team pioneered the first clinical trials of orthotopic liver transplantation (LT) in three patients (1). This groundbreaking procedure offered a new lease on life for patients with severe liver conditions, improving their lifespan and quality of life. The procedure’s advantages include the liver’s remarkable regenerative ability, which minimizes donor risk, and an overall increase in population survival rates. However, LT is not without its drawbacks, including surgical complications, high costs, and the requirement for lifelong immunosuppression. Above all, the chronic shortage of available organs has been a persistent hurdle, underscoring the need for more feasible treatment alternatives.

### 1.2 The advent and progress of hepatocyte transplantation

**Figure 1:**
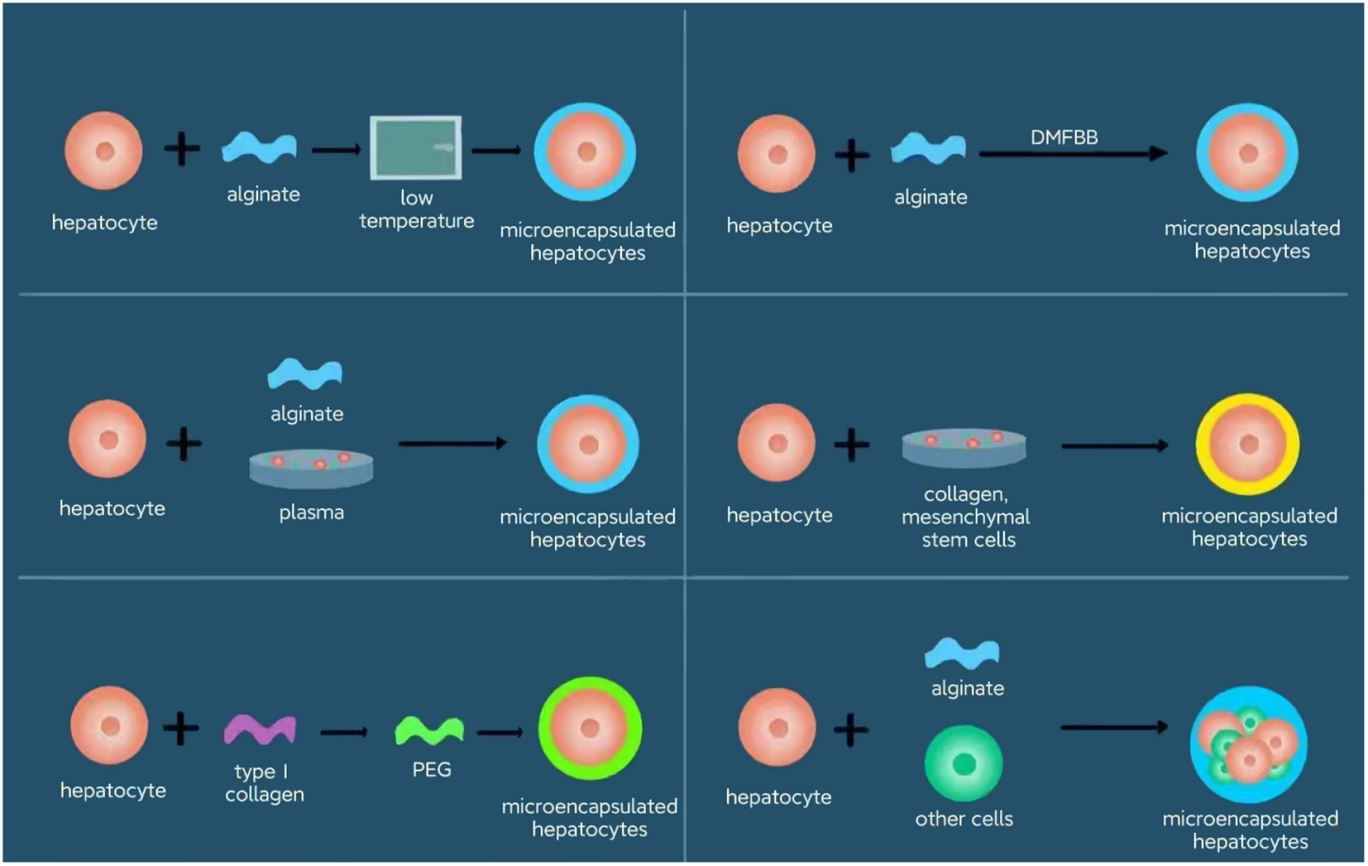
Encapsulation approaches for hepatocytes. The various encapsulation methods discussed herein are summarized, providing a concise overview of the innovative approaches in hepatocyte transplantation. The approaches include alginate plus low temperature, alginate plus plasma, type 1 collagen and PEG, alginate plus diversion-type microcapsule-suspension fluidized bed bioreactor (DMFBB), collagen plus mesenchymal stem cells, and alginate plus other cells.

The growing discrepancy between the demand for liver transplants and the available supply, as highlighted in recent reports by the Organ Procurement and Transplantation Network (OPTN) and the Scientific Registry of Transplant Recipients (SRTR), underscores the urgent need for alternative liver tissue sources. Hepatocytes, or liver cells, possess distinct characteristics that make them particularly appealing for transplantation; they retain functional capabilities even when isolated, and cryopreserved hepatocytes can be rapidly deployed for urgent therapeutic needs. This realization sparked interest in the potential of hepatocyte transplantation (HT) as a viable alternative to LT for managing LBMD and acute liver failure (ALF). Although hepatocyte transplantation is limited by many obstacles in clinical practice, researchers are constantly working to overcome them. Tasks remains to solve include scarce supply of reliable and high-quality hepatocytes, sub-optimal survival and regeneration after hepatocyte transplantation with transient phenotype, and urgent need of more effective immunosuppressive protocols to reduce rejection (2). Demonstrated to functionally mimic the liver to a certain extent, especially in acute cases, HT presents several advantages over traditional LT. One donor liver could potentially benefit multiple patients, depending on the yield of viable cells obtained and the specific needs of each patient (3). HT offers a less invasive approach compared to LT, eliminating the need for major surgery. Additionally, the ability to repeat hepatocyte infusions and preserve cells for future use means that patients on the liver transplant waitlist can maintain some liver function until a suitable organ match is found. Furthermore, the lower costs, reduced risks, and fewer complications associated with HT have contributed to its growing popularity as a promising treatment alternative. The various encapsulation methods discussed herein are summarized in **Figure 1**, providing a concise overview of the innovative approaches in hepatocyte transplantation.

## 2 Advancements in encapsulation materials for hepatocyte transplantation

### 2.1 Factors and mechanism related

Hepatocyte Transplantation demonstrates several important factors. The location of encapsulated hepatocytes within recipients can influence their viability and functionality. Existing literature and experiments suggest that the implantation site plays a crucial role in the success of hepatocyte transplantation. For instance, encapsulated hepatocytes transplanted into the peritoneal cavity may benefit from the rich blood supply and immune-privileged status, which could enhance cell survival and function (4). The microenvironment at the transplantation site, including factors like oxygenation and nutrient availability, can significantly impact the encapsulated hepatocytes’ ability to maintain their liver-specific functions.

The dose or number of transplanted hepatocytes is a critical factor affecting transplant efficiency and the therapeutic outcome, which together with the place of transplantation, will largely decide the condition of hepatocytes (5). Determining the optimal number of hepatocytes for transplantation remains a challenge and is subject to ongoing research. The efficiency of transplantation and subsequent liver function recovery is likely dose-dependent, requiring a balance between sufficient cell mass for therapeutic effect and the host’s capacity to integrate and support the transplanted cells. Studies have suggested that a higher number of transplanted hepatocytes may improve the functional recovery in liver failure models, but this must be balanced against the risk of potential complications such as portal hypertension or embolization (6). Future research should aim to establish standardized protocols for dosing and to explore the mechanisms underlying dose-dependent effects on transplantation outcomes.

The detailed discussion on the underlying mechanisms of encapsulated hepatocytes is limited. Potential mechanisms behind the improved viability and function of encapsulated hepatocytes involve several aspects, including enhanced protection from immunological rejection, improved microenvironmental control within the capsules, and the supportive effects of co-encapsulated cells (7). For instance, encapsulation materials like alginate provide a semi-permeable barrier that can protect hepatocytes from the host’s immune response while allowing the exchange of nutrients and metabolic products (8). Additionally, co-microencapsulation with supportive cell types, such as mesenchymal stem cells, may provide trophic support and promote a more physiologically relevant microenvironment that enhances hepatocyte function (9).

### 2.2 Addressing immune rejection challenges

Addressing challenges such as immune rejection after hepatocyte transplantation, encapsulation emerges as a straightforward, economical, and effective strategy. The critical aspects of this approach—material selection, encapsulation technique optimization, and culture environment adaptation—are key to successful hepatocyte encapsulation. The choice of encapsulation material is particularly crucial as it directly impacts the encapsulated hepatocytes’ functionality and viability by influencing oxygen and nutrient transfer.

### 2.3 Alginate’s role in hepatocyte microencapsulation

Alginate, a material favored for its biocompatibility, ease of gel formation, and unique physicochemical properties, stands out in the realm of hepatocyte microencapsulation. Miranda et al. observed that alginate-encapsulated hepatocyte aggregates exhibited significantly enhanced albumin production, urea synthesis, and enzymatic activities such as 7-ethoxycoumarin O-deethylase and uridine diphosphate glucuronosyltransferase (UGT) compared to non-encapsulated controls (8). Notably, these encapsulated hepatocytes demonstrated improved functional outcomes when cultured in a bioreactor system, maintaining performance over three weeks.

In a novel exploration, Nhu-Mai et al. revealed that alginate hydrogel could shield human hepatoma-derived cells (Huh-7), the most commonly used cell line recently with high permissiveness, from Hepatitis C Virus (HCV) infection (10). This protective effect, dependent on the concentration and duration of culture, suggests alginate hydrogel’s broader viral defense capability, irrespective of encapsulation.

### 2.4 Optimization and comparative studies

Further research by Lan et al. compared the survival and metabolic function of hepatocytes encapsulated in different alginate compositions, SLM100 and SLG100, demonstrating sustained viability, enzyme secretion, and antioxidant activity under 3D culture conditions, albeit with reduced proliferation rates (11). Saeed Azandeh et al. investigated the impact of alginate hydrogel concentrations on Human Wharton’s Jelly-derived Mesenchymal Stem Cells (HWJ-MSCs) (12). They discovered that a 1.5% alginate concentration was more conducive to cell proliferation and urea production than a 2.5% concentration, highlighting the importance of finding the optimal alginate concentration for hepatocyte viability. Jitraruch et al. proposed an optimized protocol for producing alginate-encapsulated human hepatocytes, which demonstrated superior mechanical stability and ideal bead size for enhanced cell viability (13). Similarly, Pasqua et al. developed a technique for culturing hepatocytes in 1.5% alginate beads, facilitating the autonomous formation of spheroids with maintained liver functions over two weeks (14). Durkut et al. evaluated the viability and metabolism of primary rat hepatocytes encapsulated in various matrices and subjected to different cryopreservation conditions (15). Their findings indicated that cryopreservation in liquid nitrogen (LN2) best preserved hepatic functions and viability, with ACA-encapsulated hepatocytes maintaining nearly 90% of their metabolic activity post-thaw. These studies collectively underscore the significance of material selection and encapsulation conditions in enhancing the therapeutic potential of hepatocyte transplantation, paving the way for innovative approaches to liver disease treatment. The quest to mitigate immune rejection reactions post hepatocyte transplantation has steered research towards hepatocyte encapsulation as a viable, cost-effective solution. Key to this endeavor is the meticulous selection of encapsulation materials, microencapsulation methodologies, and the fine-tuning of the culture environment, all of which are pivotal for the successful encapsulation of hepatocytes.

### 2.5 Novel encapsulation materials and techniques

Stephanie H. Capone and associates explored various material combinations for cell microencapsulation, including alginate alone, alginate combined with type I collagen, with or without poly-L-lysine and alginate coatings (16). They discovered that incorporating collagen and polylysine enhanced the mechanical resilience of the beads but compromised vitamin B12 mass transfer kinetics. Alginate-collagen beads notably enhanced HepG2/C3A viability with increased metabolism rate. Upon subcutaneous implantation in mice, they also mitigated inflammation, spotlighting the crucial balance between mechanical strength, cell behavior, and biocompatibility. Subhas C. and his team innovated silk sericin–alginate–chitosan microcapsules, creating a sericin and alginate microbead core with a chitosan outer shell (17). These microcapsules, characterized by their spherical shape and glossy surface, demonstrated high cell viability and uniform encapsulation under confocal microscopy, indicating an optimized living microenvironment for the encapsulated cells.

### 2.6 Cell source considerations for clinical transplantation

A significant challenge in hepatocyte transplantation research has been sourcing cells that are both functional and safe for clinical use. Traditionally, hepatocellular carcinoma (HCC) cell lines such as HepG2, HepaRG, and HepG2/C3A have been extensively utilized in research due to their ease of propagation and maintenance. However, these cells are derived from liver cancers and, as such, are not suitable for clinical transplantation purposes. Their immortal nature, potential for uncontrolled proliferation, and inferior functionality compared to primary hepatocytes limit their applicability in therapeutic contexts. Recognizing these limitations, the field is increasingly turning towards human pluripotent stem cells (hPSCs) as a potential source of hepatocyte-like cells (HLCs) for transplantation. hPSCs, including both embryonic stem cells (ESCs) and induced pluripotent stem cells (iPSCs), possess the capacity for unlimited self-renewal and the potential to differentiate into any cell type, including hepatocyte-like cells. This differentiation is achieved through the mimicking of liver development stages *in vitro*, leading to the generation of cells that exhibit key hepatocyte functions such as albumin secretion, urea production, and drug-metabolizing enzyme activity.

The use of hPSC-derived HLCs presents a promising avenue for overcoming the cell source limitation in hepatocyte transplantation. These cells could provide a renewable, ethically accessible, and potentially customizable source of hepatocytes for therapeutic applications. Furthermore, advancements in differentiation protocols and three-dimensional culture systems are enhancing the functional maturation of HLCs, bringing them closer to the functionality of primary human hepatocytes. However, challenges remain in the clinical application of hPSC-derived HLCs, including ensuring the efficiency and consistency of differentiation protocols, the purity and safety of the cell populations (e.g., eliminating the risk of tumorigenicity), and the long-term functionality and integration of the transplanted cells *in vivo*. Addressing these challenges requires ongoing research and collaboration between stem cell biologists, tissue engineers, and clinical researchers. While HCC cell lines have provided valuable insights into liver biology and disease, the transition towards clinical transplantation necessitates the exploration of alternative cell sources like hPSC-derived HLCs. The promise of these cells in providing a viable, ethical, and functional source for hepatocyte transplantation underscores the importance of continued research and development in this exciting area of regenerative medicine.

### 2.7 Enhancing Liver-specific functions

Meng Tian et al. developed Galactosylated alginate (GA)–chitosan oligomer microcapsules, adjusting membrane porosity and thickness to balance mechanical stability and permeability (18). This selective permeability effectively transported human serum albumin while blocking immunoglobulin G, enhancing liver-specific functions within the microcapsules. In the study by Ying He et al., Cytodex 3 microcarriers formed the core of the microcapsules, enveloped by an alginic acid-chitosan-alginate (ACA) polyelectrolyte layer (19). Utilizing an aqueous two-phase emulsification technique, L02 cells on Cytodex-3 microcarriers were encased within a thin conformable layer, facilitating equitable transport of nutrients and wastes. These microcapsules consistently produced urea and human albumin *in vitro* and demonstrated the capability to stabilize serum markers in acetaminophen-damaged rats post-transplantation. Christian Siltanen et al. utilized a coaxial flow-focused droplet microfluidics approach to craft microcapsules with liquid cores and polyethylene glycol (PEG) shells (20). This encapsulation facilitated rapid aggregation of primary hepatocytes into dense globules, preserving liver function leaped from normally 1-2 days to 10 days. The technique also offered the flexibility to tailor the mechanical properties and permeability of the gel, making it adaptable for further experimental investigations. Shahla Khodabakhsh Aghdam et al. explored the incorporation of galactosylchitosan (GC) and collagen (COL) into alginate microcapsules, subsequently coated with chitosan to produce alginate-galactosylated chitosan-collagen/chitosan (AGCCol/C) microcapsules (21). This addition significantly influenced the hydrogels’ physical properties, enhancing the proliferation of HepG2 cells, and up-regulating the expression of P450 and albumin mRNA, demonstrating improved biocompatibility. anhong Zhang et al. adopted a one-step spray method to fabricate microcapsules using hyaluronic acid (HA)/sodium alginate (SA) as the core and chitosan (CS) as the shell (22). This method not only ensured high viability of C3A cells *in vitro* but also enhanced urea and albumin synthesis, highlighting HA’s role in promoting CYP450 gene expressions. Such advancements suggest a promising direction in hepatocyte microencapsulation techniques for liver transplantation applications. Further details and comparative analyses of these methodologies are summarized in **Table 1**, offering a comprehensive overview of the state-of-the-art in hepatocyte microencapsulation. Hyaluronic acid (HA) has been observed to enhance the expression of genes related to the cytochrome P450 family after a duration of three days. Utilizing an encapsulation technique, it was noted that the functionality of hepatocytes was markedly preserved, providing an improved habitat conducive to their survival. This method is seen as a potential option for the transplantation of hepatocyte microcapsules. Chan et al. explored an alternative approach by employing Double-emulsion (DE) droplets to generate microencapsulated homotypic or heterotypic hepatocyte spheroids within an alginate-collagen composite hydrogel, moving away from the sole use of alginate (42). Their microfluidics-based technique, which eliminates the necessity for spheroid loading and allows for the control over spheroid characteristics, has shown to enhance hepatocyte performance. This includes increased albumin and urea secretion, as well as improved cytochrome P450 activity. Moreover, hepatocyte function was further enhanced when co-cultured with endothelial progenitor cells at an optimal ratio of 5 to 1 in alginate-collagen. In contrast, Lee et al. experimented with hybrid hydrogels of varying stiffness to encapsulate HepaRG cells, either individually or with support cells. They utilized tissue engineering approaches to fabricate three-dimensional (3-D) liver models *in vitro* (43). When the elasticity of these 3D liver models was adjusted to closely match the range of 2.3-5.9 kPa, there was a notable increase in hepatic gene expression, albumin secretion, cytochrome p450-3A4 activity, and drug metabolism capabilities. This model also demonstrated the ability to extend the viability and functionality of hepatocytes over extended culture periods. Further contributing to this field, Cui and colleagues demonstrated that utilizing gelatin methacryloyl (GelMA) hydrogel as a base for constructing 3D lobule-like microtissues offers advantages for hepatocyte functionality (44). The GelMA hydrogel, shaped by a digital micromirror device (DMD)-based microfluidic channel, allows for the encapsulation of hepatocytes within micromodules featuring a central radial-type hole. After prolonged co-culture, hepatocytes encapsulated alongside fibroblasts showed an increase in albumin secretion and maintained over 90% cell survival rate. Chang et al. opted for volvox sphere microbeads to encapsulate hepatocytes, providing a dual-layer three-dimensional environment for the cells (45). This innovative approach contributes to the growing body of research focused on improving hepatocyte culture methods and transplantation strategies. Dynamic bioreactor cultures of AML12 hepatocytes together with rat mesenchymal stem cells (MSCs) demonstrated significant enhancements, with MSCs evolving into hepatocyte-like cells, doubling albumin (ALB) secretion, and increasing cytokeratin 18 expression by 2.5 times. In models of CCl4-induced liver damage in rats, encapsulation of MSCs and hepatocytes within volvox spheres markedly reduced AST and ALT levels, aiding liver repair and new tissue formation (46). Kim’s investigation into a three-dimensional heparin-based hydrogel scaffold for hepatocyte culture revealed that such encapsulated hepatocytes maintained high-level functionality, including albumin and urea synthesis, for up to three weeks. The addition of hepatocyte growth factor (HGF) into the hydrogel further enhanced these synthesis processes (47).

**Table 1:** Co-encapsulated cells and their results in recent studies on co-encapsulated cells.

Year	Cell types	Donor	Recipient	Transplantation site	Results	Reference
2002	hepatocyte + bone marrow stem cell	Male Wistar rats (250-300 g)	Wistar male rats, 200-250 g	in vitro and in vivo (intraperitoneal)	improve the viability and feasibility of liver	(23)
2004	hepatocyte + bone marrow stem cell	Male SD rate (150-200 g)	not found	in vitro	improve the urea synthesis and albumin secretion activities	(24)
2004	hepatocytes +islets	Hepatocytes: Male Wistar rats (150 g); islets: Male Wistar rats (250 g)	Kunming mice (30-36 g)	in vitro and in vivo (intraperitoneal)	albumin level better maintained, BG sooner return to normal	(25)
2007	hepatocytes+human umbilical vein endothelial cells	hepatocytes: a subclone of rat hepatoma HIIE cells from Prof. K. Motojima, Meiji Pharmaceutical University, Tokyo, Japan; HUVECs: Toyobo Co., Ltd. (Osaka, Japan)	not found	in vitro	increase some genes expression caused by cell to cell communication	(26)
2003	hepatocyte+bone marrow stem cell	Male Wistar rats (200-250 g)	homozygous gunn rats, j/j, 200g	in vitro and in vivo (intraperitoneal)	maintain function in vitro and improve bio-ability in vivo	(27)
2008	rat hepatocytes + mouse fibroblast, NIH/3T3 cell	Male Wistar rat aged 5-8 weeks	not found	in vitro	promote cell growth and maintain all sorts of functions	(28)
2009	hepG2 +sertoli cells	not found	not found	in vitro	better function and bioactivity	(29)
2009	hepatocyte _bone marrow mesenchymal stem cell	SD rats (180-200 g)	SD rats (180-200 g)	peritoneum	improve albumin secretion and urea synthesis	(30)
2005	HepG2+sertoli cells	sertoli cell: Male Wistar rats, age 15-20 days; HepG2 cells: ECACC (Wiltshire, UK)	Young male Wistar rats	in vitro and in vivo (intraperitoneal)	protect cell with locally generated immuno-suppression	(31)
2010	rat hepatocyte_human fetal liver stromal cells	Wistar rats (160-180 g) and Balb/c mice (22-25 g)	Balb/c mice	in vitro and in vivo (peritoneum)	improve the survival of acute liver failure rat model and maintain function	(32)
2014	hepatocyte +adipose-derived stem cells	Female SD rats (180-200 g)	female SD rats (180-200 g)	in vitro and in vivo (peritoneum)	better functions and viability	(33)
2015	hepatocyte+human mesenchymal stem cells	human hepatocyte: reject or unuse for orthotopic liver transplantation or liver resections at King's College Hospital (London, UK); MSCs: human umbilical cord matrix	not found	in vitro	enhance urea synthesis and albumin secretion and improve the viability	(34)
2015	iPS-human+stem cells	iPS-H: laboratory of Dr. Stephen Duncan at the Medical College of Wisconsin; SCs: Dr. Howard Green at Harvard Medical School	C57BL/6 mice	in vitro and in vivo (intraperitoneal)	improve human albumin and α1-antitrypsin (A1AT)	(35)
2018	porcine hepatocyte+human mesenchymal stem cells	porcine hepatocyte: 10 kg pigs (M. Stirnimann, Apples, Switzerland); human MSCs: femoral head of the patients undergoing total hip replacement	not found	in vitro	day 3: possess albumin secretion and diazepam catabolism; day 4 and 8: better buo-activity and longer span of albumin secretion	(36)
2018	hepatocyte+mesenchymal stem cells	hepatocytes: reject or unuse for orthotopic liver transplantation; MSCs: Wharton's jelly (cords from C-sections)	Male SD rats 8-10 weeks (200-300 g)	in vitro and in vivo (intraperitoneal)	improve behavior and function	(37)
2018	hepatocyte +human umbiilical vein endothelial cells	hepatocyte: according to seglen; HUEVCs: ATCC	SD rats (180-220 g)	in vitro and in vivo (intraperitoneal)	maintain hepatocyte-specific function	(38)
2020	primary human hepatocytes+collagen fibroblasts	hepatocyte: 54-year-old female Caucasian, 16-year-old female AsianCollagen I: Corning Life Science, Tewksbury, MA, USA)	not found	in vitro	improve hepatic functions and gene expression	(39)
2020	hepatocytes+HNF4α-overexpressing human umbilical cord MSCs	hepatocyte: patients who undergoing partial hepatectomy or liver transplantation; UMSCs: Wharton's jelly of umbilical cord	Male C57BL/6 mice (8-10 weeks)	in vitro and in vivo (intraperitoneal)	produce therapeutic effect	(40)
2021	HepLPCs+HUVECs	according to previous study	NSG mice	in vitro and in vivo (intraperitoneal)	alleviate liver injury caused by CCL4	(41)

Gevaert et al. compared HepG2 cell encapsulation effects between galactosylated gelatin and Methacrylamide modified gelatin, finding that methacrylamide modification had little impact on viability, whereas galactosylated gelatin significantly enhanced specific gene expression over long-term culture (>21 days) (48). Lee and co-researchers discovered that hepatic function in primary human hepatocytes (PHHs) encapsulated within biodegradable hydrogel systems was best maintained with hydrogels of intermediate initial degradability, outperforming Matrigel in cytochrome P450 functional assays (49). Wang et al. introduced a one-step synthesis method for creating composite hydrogel capsules (CHCs) characterized by uniformity, biocompatibility, stability, and high-throughput capabilities, showing that hepatocytes encapsulated in CHCs exhibited enhanced viability, growth, and liver-specific functions (50). Tirella’s study presented a protein/hydrogel formulation as a novel encapsulation choice, enhancing nutrient exchange and providing a 3D adhesive framework for cells. This study also included encapsulating ratiometric optical nanosensors within hepatocytes to monitor microenvironmental pH changes under stress, noting improved albumin secretion and urea production in encapsulated hepatocytes compared to controls (51). Khanal et al. developed a method for creating polymeric nanofiber-integrated alginate (PNA) hydrogel microcapsules using a Nano-in-micro (NIM) system, with PNA-10 showing optimal support for HepG2 cell growth and maintenance of liver-specific metabolic functions (52). Zheng and colleagues’ research on self-bonding real-time shape-programmable microcapsules via photo-induced electrodeposition (PIED) of cell-laden alginate hydrogel found that pre-coating with fibroblasts led to robust assembly through fibroblast-ECM interactions, closely mimicking tissue morphogenesis. HepG2 cells encapsulated in these new microcapsules showed nearly double the albumin and urea secretion compared to non-fibroblast-coated encapsulations (53). Yu et al. experimented with microcapsules of various inner structures and deformability, finding that hepatocyte viability was consistent across different types, but cell activity was significantly reduced in capsules with lower deformability (54). Cui’s work on spatially assembling gear-like microstructures from photo-crosslinkable poly (ethylene glycol) diacrylate (PEGDA) hydrogel, which co-encapsulated hepatocytes and fibroblasts, resulted in 3D lobule-like micro-architectures with high cell viability and proliferation, significantly enhancing albumin secretion and urea synthesis (55). Liu and colleagues’ study on encapsulated rat liver (RLC-18) cells forming hepatic lobule-shaped microtissue (HLSM) reported superior hepatic-specific functions in these structures compared to normal cell spheroids after 14 days of culture in poly-L-lysine-alginate microcapsules (56). Moriyama et al. developed a method for producing hydrogel microbeads using an octa-thiolated PEG derivative (8-arm PEGSH), which maintained higher levels of specific functions including albumin secretion and urea production when HepG2 cells were encapsulated (57). Agarwal and team’s application of decellularized Caprine liver ECM (CLECM) derived hydrogel for 2D and 3D hepatocyte cultures showed significantly enhanced functions, including albumin, urea, glycogen, and GAGs synthesis, and the formation of bile canaliculi-like structures and better expression of mature hepatocyte markers compared to collagen coatings (58). Zhang et al. investigated the effects of 2-(1’H-indole-3’-carbonyl)-thiazole-4-carboxylic acid methyl ester (ITE) on Huh7 cells/C3A cells in both monolayer cultures and microspheres, noting significant improvements in protein levels and metabolic activities of major cytochrome P450 enzymes (59). Ravichandran et al. explored two methods to generate photocrosslinkable methacrylated liver extracellular matrix (LivMA) hydrogels, finding better cytocompatibility for encapsulated hepatocytes despite different mechanical properties (60). Zhang and colleagues compared hepatocytes encapsulated in lupeol liposomes and Gal-lupeol liposomes, with the latter showing higher cell-uptake and apoptotic efficiency in HepG2 cells, along with reduced expressions of AKT/mTOR-related proteins and markers *in vitro* and *in vivo*, demonstrating liver targeting effects in FVB mice (61). Leroux introduced a novel hybrid alginate microcapsule using an aqueous stable titania precursor (TiBALDH) and a cationic polyamine (PDDAC), leading to increased mechanical stability and maintained hepatocyte functions for up to 43 days (62). Sk’s novel synthesis of photo-crosslinkable glycidyl methacrylate (GMA) functionalized gelatins (Gelatin-GMA) enhanced cell growth and cellular functions in Huh-7.5 cells encapsulated in 3D hydrogel scaffolds.

The cultivation environment’s materials for microencapsulated cells significantly impact cell function preservation. Tostoes et al. discovered that liver-specific functions such as urea production, phase I drug metabolizing activity, and oxygen uptake in hepatocytes encapsulated within ultra-high viscous alginate spheroids were substantially improved under a continuous perfusion system compared to a traditional 50% medium change routine, tripling the performance. However, albumin output remained consistent across both feeding methods (63). Sofia P. et al. proposed a three-dimensional culture strategy for HepaRG cells in alginate microcapsules without dimethyl sulfoxide (DMSO), enhancing hepatocyte differentiation significantly over 2D cultures. This approach yielded a higher prevalence of hepatocyte-like over biliary-like cells, alongside improved protein secretion and ammonia detoxification, despite some variance in basal gene expression levels (64).

### 2.8 Challenges of translating encapsulation materials to clinical use

The diversity of materials used for the microencapsulation of hepatocytes presents a spectrum of opportunities and challenges for clinical translation. Among these, photo-crosslinkable methacrylate-based materials have gained attention for their versatility, tunability, and the precision with which they can be manipulated using light. However, translating such advanced materials into clinical use encompasses several hurdles, particularly concerning safety, efficacy, and regulatory approval.

#### 2.8.1 Safety and biocompatibility

The primary concern with any biomaterial intended for clinical use is its safety and biocompatibility. Photo-crosslinkable methacrylates, while useful in creating stable and customizable encapsulation systems, must be rigorously tested to ensure they do not elicit adverse immune responses, cause inflammation, or release toxic degradation products within the body. Long-term biocompatibility studies are essential to assess the risks of using these materials in humans.

#### 2.8.2 Degradation and clearance

Understanding the degradation behavior of methacrylate-based materials *in vivo* is crucial. The materials must degrade at a rate that is compatible with tissue healing and regeneration processes without causing obstruction or toxicity. Moreover, the degradation products must be safely metabolizable or excretable by the human body.

#### 2.8.3 Regulatory approval

Gaining regulatory approval for new biomaterials can be a complex and lengthy process. Regulatory bodies, such as the U.S. Food and Drug Administration (FDA) and the European Medicines Agency (EMA), require comprehensive data on the manufacturing process, quality control, safety, and efficacy of the biomaterials. For photo-crosslinkable methacrylates and other novel encapsulation materials, demonstrating compliance with these requirements involves extensive preclinical and clinical testing.

#### 2.8.4 Scalability and consistency

Translating laboratory-scale encapsulation processes to clinical-scale production presents challenges in ensuring scalability, consistency, and cost-effectiveness of the material synthesis and encapsulation procedures. Ensuring that the properties of photo-crosslinkable methacrylates remain consistent across batches is critical for maintaining the reliability of the encapsulation system.

#### 2.8.5 Ethical and legal considerations

The use of synthetic materials in medicine also raises ethical and legal considerations, particularly regarding long-term outcomes and patient consent. Patients must be fully informed of the benefits and risks associated with the use of such materials in treatments. While photo-crosslinkable methacrylate-based materials and other innovative encapsulation strategies offer significant potential for enhancing hepatocyte transplantation therapies, their path to clinical application is paved with challenges. Addressing these requires a multidisciplinary effort, combining insights from materials science, biology, medicine, and regulatory science to ensure that the benefits of these advanced materials can be safely and effectively realized in clinical settings.

### 2.9 Future directions in hepatocyte encapsulation

Despite the promising attributes of embryonic stem cells, such as high proliferation, renewability, and pluripotency, their differentiation into hepatocytes faces technical challenges, requiring an optimal culture microenvironment. Tim Maguire et al. explored how an alginate-based microenvironment supports cell viability, promotes differentiation, and enhances the functionality of embryonic stem cell-derived hepatocytes, demonstrating urea and albumin synthesis as key functional indicators. This finding suggests a viable alternative for sourcing human hepatocytes for transplantation (65). MacPherson and colleagues designed a 3D scaffold from a non-fibrous hydrogel, emphasizing mechanical properties and nanofiber morphology to enhance hepatocyte culture. Their findings showed stable maintenance of primary human hepatocytes’ viability and functionality, outperforming Matrigel in cytochrome P450 assays (66). Chen et al. assessed the stability of various alginate-based microcapsules in plasma, finding that alginate-α-poly (L-lysine)-alginate (α-APA) microcapsules demonstrated superior stability over alginate-ϵ-poly (L-lysine)-alginate (ϵ-APA) and alginate–chitosan–alginate (ACA) capsules. The stability of these capsules was influenced by different factors, with heparin significantly affecting α-APA microcapsules, while HCO3- and H2PO4-/HPO42- impacted ϵ-APA and ACA capsules, respectively (67). Liu introduced a method for creating porous alginate beads (PABs) using an aqueous two-phase system (ATPS) emulsion technique, blending a cell/dextran (Dex) mixture with an alginate (Alg)/polyethylene glycol (PEG) mixture. This approach allowed for control over the pore size, improving cell activity, proliferation, and function of encapsulated HeLa and human liver cancer cells compared to those in general alginate beads (GABs) (68).

Shogo Nagata et al. devised a technique for encapsulating cells within nucleocapsid hydrogel microfibers, creating a fibrous 3D ECM-rich microenvironment suitable for *in vitro* liver tissue formation. Induced pluripotent stem cell-derived hepatocytes (iPSC-hepatocytes) in this setup displayed liver-specific characteristics, including albumin secretion and liver marker gene expression, and maintained structural stability, indicating their potential for liver failure rescue. Transplantation of these microfibers into immunodeficient mice showed human albumin presence in peripheral blood after three days, confirming their viability and function as implants (69). Further details are presented in **Table 1**.

## 3 Enhancing hepatocyte viability and functionality

Ensuring high activity levels in hepatocytes is crucial for their ability to substitute for failing liver functions. However, hepatocytes are notably delicate, with even minor damages potentially leading to cell death and loss of activity. For microencapsulated liver cells to fulfill their intended roles post-transplantation, preserving their viability and functionality becomes a critical concern.

### 3.1 Hepatocyte cryopreservation

The primary strategy for the long-term storage of microencapsulated hepatocytes is cryopreservation. The effectiveness of this method and the ability of hepatocytes to resume their functions upon thawing are areas of active research. Mai and colleagues demonstrated that primary rodent hepatocytes could retain their synthetic functions temporarily through encapsulation and cryopreservation as early as 2005 (70). Despite the influence of immortalization on certain hepatocyte-specific functions remains questionable, which is to remove the upper limit of cell proliferation set by telomerase by either gene reactivation or deactivation, their findings suggested that both naïve and genetically modified hepatocytes could maintain metabolic functions and improve survival rates in xenogeneic recipients with liver failure when encapsulated, cryopreserved, and then transplanted, marking a significant advancement in hepatocyte therapy.

A subsequent study by Hang focused on the functional recovery of hepatocytes after cryopreservation (71). Results showed that pre-incubation at 4°C for 12–24 hours, followed by encapsulation in alginate–poly-L-lysine–alginate microcapsules, significantly enhanced hepatocyte functions, including mRNA and protein levels, as well as albumin and urea secretion post-thawing. The morphology and albumin production of post-thaw hepatocytes closely matched those of directly cultured groups over several days, underscoring the reliability of cryopreservation for hepatocyte storage despite potential risks to cell viability and functionality.

Kilbride et al.’s research revealed that alginate-encapsulated HepG2 liver microcapsules subjected to cryopreservation and subsequent short-term exposure to temperatures below 10°C from 1 to 90 minutes showed increased cell proliferation during 7-16 days of culture (72). This method presents a more efficient and cost-effective approach to achieving higher cell densities (73).

Recent work by Jitraruch et al. identified a pan-caspase inhibitor (ZVAD) that enhances the ultrastructure of cryopreserved hepatocyte microbeads and reduces cell apoptosis when combined with other cytoprotectants such as des-feroxamine (DFO), and human serum albumin (HSA) in the cryopreservation process (74). This improved cryopreservation technique optimizes the use of hepatocytes for emergency applications.

### 3.2 Alternative strategies for sustaining hepatocyte function

The liver’s metabolic capacity is immense, capable of processing nutrients as well as detoxifying substances and drugs. Koizumi et al. were the first to demonstrate that primary rat hepatocytes retain their drug metabolism and transport activities post-cryopreservation when encapsulated (75). Activities of a specific drug-metabolizing enzyme (CYP3A2) and drug transport for several substrates were maintained up to 120 days using a novel cryopreservation technique developed by the researchers.

Encapsulation aims to shield hepatocytes from the host’s immune system, yet the release of bioactive molecules from hepatocytes can potentially trigger immune responses (31). The extent of this reaction depends on the encapsulation material’s permeability and the host’s sensitivity. In animal studies, Baldini et al. showed that long-term cryopreserved encapsulated porcine hepatocytes maintained significant activity and viability when transplanted into rats without immunosuppression (4). Although the ultrastructure and morphological activity of encapsulated hepatocytes were maintained post-explant, albumin synthesis was adversely affected, indicating a need for further improvements in maintaining bio-activity post-transplantation. Maximizing the use of donor livers involves isolating hepatocytes for propagation, making the retention of their functional capabilities post-isolation critically important.

Another investigation intended to test efficacy of bioartificial liver device in 2014. In this investigation, the evaluation of Alginate-chitosan microencapsulated hepatocytes’ bioactivity was based on several metrics: cell proliferation, efficiency in ammonia detoxification, albumin production, and the rate of diazepam metabolism. The findings highlighted that, with the exception of cell proliferation which remained constant, immortalized human hepatocytes (HepLL) groups demonstrated superior performance in ammonia detoxification, albumin production, and diazepam metabolism compared to the HepG2 groups across all time points (29). Additionally, the viability of hepatocytes in spinner cultures showed variability over time, with day 10 marking the peak of cell growth, metabolic activity, and functionality.

Yamada et al. introduced a culture and encapsulation technique utilizing a Thermo-reversible gelation polymer (TGP), which transitions from solid to liquid states with temperature changes. This study revealed that hepatocytes encapsulated in TGP maintained over 70% viability after being cryopreserved in liquid nitrogen. Post-transplantation into the rat spleen, these hepatocytes were capable of performing liver-specific functions and secreting albumin (23).

Li and colleagues devised a method for hepatocyte encapsulation that involved micropatterning on collagen I to direct cell–cell interactions in two dimensions, followed by the formation of stable aggregates through collagenase digestion for three-dimensional encapsulation in polyethylene glycol (PEG) diacrylate. This configuration preserved the encapsulated hepatocytes’ specific functions for up to 50 days (27).

Lu and associates developed an innovative diversion-type microcapsule-suspension fluidized bed bioreactor (DMFBB), offering several enhancements over the traditional fluidized bed bioreactor (FBB), especially under conditions of high perfusion velocity. The research noted a significant reduction in the void volume of alginate/chitosan microcapsules and lower damage rates during the fluidization process in the DMFBB. It was observed that encapsulated C3A cells exhibited higher survival rates and activities of CYP1A2 and CYP3A4 in the DMFBB, though improvements in albumin and urea synthesis were modest. Additionally, there was a notable upregulation in the transcription levels of various CYP450-related genes and an albumin-related gene in C3A cells within the DMFBB (24).

### 3.3 Enhancing hepatocyte function through co-microencapsulation

As hepatocyte transplantation emerges as a viable option for treating acute liver failure, the sustained activity and functionality of individual hepatocytes until the point of liver transplantation remain challenges. Recognizing that the liver comprises not only hepatocytes but also non-parenchymal derivatives, which provide essential structural, biochemical support, and nutrients, underscores the pivotal role mesenchymal cells play *in vivo*. Their presence is crucial for supporting the physiological activities of hepatocytes. This understanding has spurred interest in the co-culture and co-microencapsulation of hepatocytes with various mesenchymal cells to augment hepatocyte survival and functionality, particularly for cell transplantation applications primarily investigated in animal models. While co-culture and co-microencapsulation differ significantly, insights from hepatocyte co-culture studies offer valuable perspectives for advancing co-microencapsulation strategies. Initial investigations by Rahman et al. demonstrated the protective effects of co-encapsulating HepG2 cells with Sertoli cells in animal models of acute hepatic failure (AHF), achieving localized immunosuppression and enhancing HepG2 cell survival post-intraperitoneal injection in rats (76). This approach suggested a novel strategy for cell transplantation, potentially reducing rejection risks by locally generating immunosuppressive environments.

Zheng et al. conducted further research to ascertain the efficacy of co-microencapsulating Sertoli cells with HepG2 cells in a rat model, aiming to establish a method of local immunosuppression facilitated by the unique immunoprivileged nature of Sertoli cells (30). Their findings indicated that such co-microencapsulation could enhance the function and bioactivity of hepatocytes in models of acute liver failure, offering a more effective solution than either mixed or solely microencapsulated hepatocytes and Sertoli cells.

Moreover, Liu and collaborators explored the potential of co-encapsulating hepatocytes with bone marrow stem cells using a novel two-step cell encapsulation technique. This method proved to enhance hepatocyte viability and support liver function in models of acute liver failure (34). Compared to traditional single-step encapsulation, this innovative approach resulted in extended hepatocyte viability beyond four months post-transplantation, with a noticeable reduction in host reaction and improved hepatocyte function due to the synergistic effects of co-encapsulation with bone marrow cells. Further investigations confirmed the superior viability and functionality of this co-encapsulation strategy both *in vitro* and *in vivo*, demonstrating its capacity to ameliorate conditions like hyperbilirubinemia in Gunn rats post-transplantation (36).

Isoda et al. identified bone marrow stromal cells (BMSCs) as another promising candidate for hepatocyte co-culture, showing significant support for differentiated hepatocyte functions, notably in enhancing urea synthesis and albumin secretion (37). Their sandwich-like co-culture model, comprising a monolayer of BMSCs, a semi-permeable membrane, and freshly isolated hepatocytes, revealed the critical role of interleukin-6 in maintaining these key hepatocyte functions.

The exploration of mesenchymal stem cells (MSCs), known for their limited self-renewal while multidirectional differentiation capabilities, has become a focal point of recent research (40). The minimal ethical and legal hurdles associated with MSCs, coupled with their easy extraction from various sources including umbilical cord, endometrial polyps, menstrual blood, bone marrow, and adipose tissue, make them a compelling option for experiments in hepatocyte co-encapsulation. Shi and colleagues highlighted the potential of bone marrow mesenchymal stem cells (BM-MSCs) in enhancing hepatocyte functionality when co-encapsulated, observing significant improvements in hepatocyte survival, liver function, and cellular changes post-transplantation using immunofluorescence microscopy. This study illustrated not only an increase in the specific functions of hepatocytes, such as albumin secretion and urea synthesis, but also an enhancement in cell cycle progression *in vitro*. Furthermore, hepatocyte transplantation strategies incorporating co-encapsulation demonstrated enhanced viability and bioactivity in rats models of acute liver failure, with MSCs potentially differentiating into hepatocyte-like cells and assuming liver metabolic functions (25).

Fitzpatrick et al. explored the advantages of coculturing human MSCs with hepatocytes, observing significant benefits in hepatocyte functions and viability when in direct or indirect contact with MSCs (26). This interaction notably increased albumin and urea production, with peak effects observed around day 15 for albumin. The study confirmed that coculturing hepatocytes with MSCs could enhance hepatocyte viability by up to 16%, suggesting a promising approach for cell transplantation.

Yang et al. developed a tissue engineering-based platform using cell-laden microbeads in a 3D printed tubular perfusion bioreactor, finding that co-encapsulation of human hepatocytes with collagen and MSCs resulted in improved cell activity and maintenance of parenchymal cell functions for up to 30 days (77). This setup facilitated better oxygen and medium diffusion, vital for sustaining cell vitality. Montanari and colleagues focused on the coculture and co-microencapsulation of porcine hepatocytes with human MSCs, identifying a beneficial role of MSCs in enhancing hepatocyte bioactivity and function (28). Their findings indicated that while hepatocyte viability may initially decrease, coencapsulation with MSCs led to sustained albumin secretion and diazepam metabolism, underlining the positive impact of MSCs on hepatocyte functionality.

Iansante et al. established a high-throughput system for cell encapsulation research, enabling the comparison of various conditions such as cell numbers, combinations, and alginate modifications (39). Their platform revealed that MSCs could notably improve the behavior and function of hepatocyte microcapsules. This enhancement was further validated through low-throughput analysis, underscoring the promising role of MSCs in boosting hepatocyte function.

Kong and colleagues demonstrated the therapeutic effects of co-encapsulating hepatocytes with HNF4α-overexpressing human umbilical cord MSCs (HNF4α-UMSCs) in models of acute liver failure (33). Their research showed that HNF4α-UMSCs could significantly enhance hepatocyte microbead functions and accelerate M2 macrophage polarization, potentially reducing the inflammatory response through the paracrine factor HB-EGF secreted by HNF4α-UMSCs. This study not only confirmed the functional benefits of co-encapsulation but also highlighted the underlying mechanisms contributing to improved outcomes in acute liver failure treatment.

Gao et al. explored co-encapsulation of hepatocytes with islets to create a bioartificial liver support system, showing a marked improvement in survival rates and biochemical parameters in ALF mice models (32). Takayama et al. discovered that co-culturing hepatocytes with human umbilical vein endothelial cells (HUVECs) enhances cellular functions, attributing this improvement to increased expression of certain genes linked to cell-to-cell communication (38). This insight into gene expression dynamics underlines the potential of co-culture systems in advancing liver tissue engineering.

Kim and colleagues developed a co-culture system based on cell sheets, demonstrating sustained albumin secretion and enhanced expression of hepatocyte-specific genes, thus significantly preserving hepatocyte functions (41). Nishikawa et al. showed that co-cultivating rat hepatocytes with NIH/3T3 fibroblasts on collagen-immobilized PDMS membranes enhances growth and function, notably albumin secretion, by providing ample oxygen (35).

Kukla et al. found that co-encapsulation of primary human hepatocytes with supportive fibroblasts significantly improves hepatic functions and gene expression, highlighting the benefits of incorporating 3T3-J2 murine embryonic fibroblasts or primary human hepatic stellate cells (HSCs) (78). Zhang et al.’s research on co-encapsulating hepatocytes with adipose-derived stem cells (ADSCs) demonstrated dramatic advantages in enhancing hepatocyte functions and viability, suggesting a potent cell-based therapy for liver failure (79). Teng and colleagues introduced a strategy employing rat hepatocytes and human fetal liver stromal cells (hFLSCs) for acute liver failure treatment, showing that the co-encapsulated approach significantly improves survival and hepatic function, partly due to the release of basic fibroblast growth factor (bFGF) (80). Qiu et al. confirmed the efficacy of co-encapsulating hepatocytes with HUVECs in treating fulminant hepatic failure (FHF), observing improved biochemical parameters and reduced mortality in rat models (81).

Liu’s study on co-encapsulating human hepatocyte-derived liver progenitor-like cells (HepLPCs) with HUVECs highlighted the potential of this approach in ameliorating liver injury in mice, facilitated by the secretion of glial cell line-derived neurotrophic factor (GDNF) from HUVECs (82). Song et al. demonstrated that co-encapsulation of human induced pluripotent stem cell-derived hepatocyte-like cells with stromal cells in hydrogel capsules maintains human albumin and α1-antitrypsin levels effectively in mouse sera, mirroring the performance of primary hepatocyte aggregates (83). Most recently, Xiang Yuan’s research on proliferating human hepatocytes (ProliHHs) revealed that Encapsulated ProliHHs could be engineered, intraperitoneally transplanted to those liver-failure animals, causing liver functions to reinforce though alleviated hyperammonemia and hypoglycemia, leading to less severe post-hepatectomy liver failure (PHLF) with minimal inflammatory response, adverse effects or tumorigenic (84).

These findings collectively underscore the vast potential of co-encapsulation strategies in enhancing hepatocyte functionality and viability, offering new avenues for liver failure treatment and tissue engineering. Further details are summarized in **Table 2**.

**Table 2:** The selection of materials for hepatocyte microencapsulation and its influence.

Year	Material	Cell type	Donor	Site	Result	Reference
2010	heparin-based hydrogel	hepatocytes	female adult Lewis rats (125-200 g)	in vitro	maintain albumin and urea synthesis	(21)
2013	alginate+type I collagen +poly-L-lysin +alginate		HepG2/C3a HCC cells	female C57BL/6 mice (7 weeks old)	in vitro and in vivo	(10)
2014	silk sericin-alginate-chitosam microcapsules	HepG2	culture in DMEM	in vitro	high cell viability and uniform cell encapsulation distribution, increase glucose consumption urea secretion rate and albumin content	(11)
2014	galactosylated alginnate (GA)-chitosan oligomer microcapsule	normal human hepatocytes, L02	CAS	in vitro	transport albumin block IgG, enhance liver function, high viability and proliferation of human hepatocytes	(12)
2014	glalctosylated gelatin/methacrylamide modified gelatin	HepG2	Prof. M. Bracke (Ghent University	in vitro	methacrylamide modified gelatin: no influence on hepatocyte viability galactosylated gelatin: improve specific gene expression >21 days	(22)
2015	protein/hydrogel	HepG2	ATCC, passage 83	in vitro	increase albumin secretion and urea production	(44)
2016	alginage-collagen composite hydrogel	Fresh primary SD rat hepatocytes	fresh hepatocyte	in vitro	increase albumin secretion, urea secretion and cytochrome P450 activity	(17)
2016	SiO2, polu(sodium-p-styrenesulfonate)(PSS), CaCO3, porous CaCO3 spheres	SMCs cells, HepG2 Cells and Ecs cells	Cell bank of CAS	in vitro	no difference in viability between three microbeads, reduce cell activity in microcapsules with lower defonmability	(47)
2016	octa-thiolated PEG derivative (8-arm PEGSH), horseradish peroxidase, a small phenolic compound (Glycyl-L-tyrosine)	HepG2 cells	National Bio-Resource Project of MEXT, Japan	in vitro	maintain albumin secretion and urea production	(50)
2017	Cytodex 3 microcarrier+alginic acid-chitosan-alginate (ACA) polyelectrolyte layer	L02 cells	GE Healthcare, UK	in vitro and in vivo (SD rats, 8 weeks, intraperitoneal)	in vivo: maintain the serum levels of total BiL, ALT and albumin in acetaminophen	(13)
2017	liquid cores+poluethylene glycol (PEG)	primary hepatocytes of female Lweis rats (125-200 g)	female Lewis rats (125-200 g) from Charles River laboratories (Boston, MA)	in vitro	allow adjustment of the mechanical properties and diffusion of the gel, maintain liver function for more than 10 days	(14)
2017	hybrid hydrogel	undifferentiated hepaRG cells	HPR101, Biopredic International, Saint Gregoire, France	in vitro	enhance hepatic gene expression, albumin secretion, cytochrome P450-3A4 activity, and drug metabolism	(18)
2017	volvox	MSCs and AML12 cells	the femurs of 3 week old SD rats	in vitro and in vivo (male 6-week-old SD rats implantation into liver)	in vitro: MSCs differentiate into hepatocyte like cell, increase albumin secretion, increase cytokeratin 18 expression; in vivo: reduce AST and ALT levels, improve repair and formation	(20)
2017	poly-L-lysine-alginate	rat liver (RLC-18) cells	culture with DMEM	in vitro	hepatic-specific function higher than normal cell spheroids	(49)
2017	2-(1'H-indole-3'-carbonyl)-thiazole-4-carboxylic acid methyl ester	Huh7 and C3A cell	CRL-10741, ATCC	in vitro	monolayer cultures: improve protein levels and the metabolic activities and the SYP450 enzymes, SYP1A1, CYP1A2, CYP3A4 and CYP1B1; on mimcrospheres: increase the protein levels and some protein activities	(52)
2018	photo-crosslinking poly)ethylene glycol) diacrylate (PEGDA) hydrogel	HepG2 and NIH/3T3 cells	ATCC	in vitro	exhibit high cell viability, proliferatio and spreading, increase albumin secretion and urea synthesis	(48)
2018	decellularized caprine liver ECM (CLECM) derived hydrogel	HepG2	NCCS, Pune, India	in vitro	for 2D hepatocyte culture: increase albumin, urea,glycogen and GAG synthesis; for 3D culture: bile canaliculi-like structure, better expression of mature hepatocyte markers	(51)
2018	hybrid alginate+aqueous stable titania precursor+cationic poluamine	HepG2 cells	culture in DMEM	in vitro	microcapsule exhibit increased mechanical stability, maintain viability, oxygen consumption, and albumin secretion for 43 days	(55)
2019	gelatin methacryloyl hydrogel	HepG2 and NIH/3T3 cells	ATCC	in vitro	co-encapsulated with fibroblasts indicate increasing albumin secretion and over 90% cell survival	(19)
2019	polumeric nanofibreintegrated alginate	HepG2 cells	ATCC	in vitro	PNA-10 was suitable for HepG2 growth, 3D PNA-10 microcapsule maintain survival and liver-specific metabolic functions	(45)
2019	pre-coated with fibroblasts into alginate hydrogel	NIH/3T3 cells, HepG2 cells	ATCC	in vitro	increase albumin and urea secretion nearly 2 times	(46)
2020	alginate-galatosylated chitosan-collagen/chitosan	HepG2 cells	Pasture Institute, Iran	in vitro	promote the proliferation and secretion of albumin and urea, up-regulate the expression of P450 and albumin mRNA	(15)
2020	PEG/HA semi-IPN hydrogels	immortalized human hepatic sinusoidal EC-SV40 cells +fibroblasts	T0056, Applied Biological Materials, Inc., Richmond, BC, Canada	in vitro	maintain function outperform matrigel in CYP450 functional assays	(42)
2020	composite hydrogel	human liver cell, human iPSCs	cell bank of CAS	in vitro	better viability, growth, liver specific function including urea synthesis and albumin secretion	(43)
2021	hyaluronic acid (HA)/sodium alginate (SA)+chitosan (CS)	C3A cells	ATCC	in vitro	high viability, HA increases synthesis of urea and albumin, and the activity of CYP1A2, CYP3A4 and CYP450	(16)
2021	photocrosslinkable methacrylated liver extracellular matrix (LivMA) hydrogels	immortalized human hepataocytes cells	professor Didier Trono from the Ecole Polutechnique Federale de Lausanne, EPFL	in vitro	indicate better cytocompatibility	(53)
2021	Lupeol liposomes/Gal-lupeol liposomes modified with Gal-PEG-DSPE	HepG2	not found	in vitro and in vivo (FVB/N mice, high pressure tail vein transfection)	Gal0-lupeol-liposome: highest cell-uptake efficiency and higher apoptotic efficiency, Gal-lupeol-liposome: reduce expression of Akt/mTOR-related proteins in vitro, AFP, GPC3, and EpCAM mRNA expression	(54)
2021	gelatin derivatives-photo-crosslinkable glycidyl methacrylate (GMA) functionallized gelatins	Huh-7.5 cells	RIKEN (VA, Japan)	in vitro	enhance cell growth, improve differentiation, viability and proliferation	(56)

## 4 The impact of xenotransplantation

With hepatocyte transplantation emerging as a notable strategy for addressing acute liver diseases, the scarcity of human liver donations has prompted the exploration of xenotransplantation. This approach, involving the transplantation of hepatocytes from other species, offers a potential solution to the shortage of allogeneic hepatocytes. However, numerous challenges, including immunological rejection and the potential for anaphylactic reactions to xenoproteins, necessitate further investigation. Studies have shown that encapsulation can enhance the survival and function of both fresh and cryopreserved porcine hepatocytes in models of fulminant liver failure (85). These studies revealed two critical phases: an *in vitro* decline in metabolic functions over a week post-transplantation and an *in vivo* extension of survival rates and maintenance of metabolic functions in encapsulated hepatocytes compared to non-encapsulated controls. Sgrio et al. discovered that encapsulated human hepatocytes, immortalized to stabilize metabolic functions, could substantially support metabolism and mitigate liver regeneration inhibition in acute liver failure models by reducing inflammatory stress (86). This dual approach highlights the need for distinct research focuses on metabolic function and regeneration in acute liver failure studies. Furthermore, encapsulated transplantation was found to significantly reduce cytokine levels, illustrating a decrease in inflammatory stress and a restraint on the regeneration of remaining hepatocytes (87).

Investigations into porcine hepatocytes as a xenotransplantation source have identified potential limitations, including safety concerns related to porcine endogenous retroviruses. Despite these challenges, studies have demonstrated therapeutic effects of encapsulated porcine hepatocytes in rodent and non-human primate models of fulminant liver failure (88). The use of neonatal pig re-aggregated liver cells (NPRLCs) has shown promise in improving survival rates and metabolic function in acute liver failure models, suggesting an alternative to alleviate the human hepatocyte shortage (89).

Machaidze et al. explored the transplantation of encapsulated miniature swine hepatocytes in baboons with fulminant liver failure, revealing a temporary support to liver metabolism and a restoration of normal liver functions in the majority of the treated animals (82). This indicates a viable method for large mammal xenotransplantation. Varaa et al. examined the effects of umbilical cord stem cells (UCSCs) and UCSC-derived hepatocyte-like cells (HLCs) encapsulated in high mannuronic alginate scaffolds on acute liver failure models, showcasing significant improvements in liver function markers (90).

Xenotransplantation research suggests that while xenohepatocytes offer a readily available solution to hepatocyte scarcity, the immunorejection challenge remains significant. Therefore, less immunogenic transgenic pigs and innovative cell encapsulation techniques are being considered as future research directions to address these hurdles (91). This overview underscores the complexities and potential of xenotransplantation in hepatocyte therapy, emphasizing the need for ongoing investigation into improving viability and functionality through advanced encapsulation methods and genetic modifications. Details on these studies are summarized in **Table 2**.

## 5 Clinical applications and future prospects

Although encapsulated hepatocyte technology has demonstrated promising results *in vitro* and in animal models, its transition to clinical applications presents a horizon ripe with potential. The clinical implications of hepatocyte encapsulation span several critical areas in liver failure treatment and regenerative medicine.

One primary application envisaged for encapsulated hepatocytes is in the development of a bioartificial liver device (BAL). Such devices aim to provide temporary liver support for patients with acute liver failure, bridging the gap to liver regeneration or transplantation. Encapsulated hepatocytes within BALs offer a biocompatible and immunoprotected environment, which could enhance cell function and longevity, thus improving the therapeutic efficacy of these devices. Moreover, the potential for allogeneic or xenogeneic cell transplantation without the need for lifelong immunosuppression could revolutionize the treatment landscape for liver diseases. The microencapsulation technique acts as a barrier to immune cells while allowing the exchange of nutrients and metabolic waste, making it a promising approach for cell transplantation therapies.

Clinical trials exploring the efficacy and safety of encapsulated hepatocyte transplantation are crucial next steps. Such studies will help determine the optimal cell sources, encapsulation materials, and transplantation protocols. Additionally, understanding the long-term outcomes of these interventions, including the risk of potential complications and the durability of treatment effects, is essential. Furthermore, integrating advances in biomaterials and stem cell technology could enhance the clinical applicability of hepatocyte encapsulation. For instance, the use of stem cell-derived hepatocytes for encapsulation could overcome the limitations associated with donor cell availability. Innovations in encapsulation materials that mimic the liver extracellular matrix could further support hepatocyte function and integration post-transplantation. In conclusion, while encapsulated hepatocytes herald a promising frontier in liver failure therapy, significant efforts in clinical research and technology development are necessary to translate these experimental approaches into viable clinical solutions. The progression of encapsulated hepatocyte technology into clinical trials and ultimately clinical practice will require multidisciplinary collaboration among scientists, clinicians, and regulatory bodies to ensure safety, efficacy, and patient accessibility.

## 6 Summary and future directions

This review has delved into the advancements in hepatocyte encapsulation research, emphasizing the strides made in preserving hepatocyte viability, the innovative approach of co-microencapsulation, the exploration of xenotransplantation, and the development of novel encapsulation materials. Hepatocyte transplantation represents a promising avenue for mitigating the immune response challenges and addressing the scarcity of liver donations, offering a beacon of hope for numerous patients. The preservation of hepatocyte activity prior to transplantation is predominantly managed through encapsulation techniques, cryopreservation, enzyme inhibitors, and immunosuppressive agents. Future investigations could enhance these methods by fine-tuning temperature controls and elucidating the roles of specific enzymes in the longevity and functionality of transplanted hepatocytes.

The strategy of co-encapsulating hepatocytes with various cell types, such as Sertoli cells, bone marrow mesenchymal stem cells (MSCs), fibroblasts, adipose-derived stem cells, and human umbilical vein endothelial cells (HUVECs), has shown to enhance the survival and functionality of hepatocyte transplants. However, the underlying mechanisms of these co-encapsulation benefits remain to be fully understood and warrant further exploration. Xenotransplantation has emerged as a viable strategy to broaden the donor pool for hepatocyte transplantation. Research has predominantly focused on encapsulating and transplanting porcine or human liver cells into models of liver failure. These preclinical endeavors have demonstrated notable improvements in the functionality of transplanted liver cells. Advancements in the materials used for hepatocyte microencapsulation have also been significant, ranging from the optimization of traditional substances like alginate to the introduction of novel materials and structures for hepatocyte microcapsules, as well as refining the pre-encapsulation cell culture environments.

Last but not least, some of the accomplishments in animal and human-cell based studies are still in need of a more cautious attitude towards clinical achievement in reality. Many experiments take advantage of the marker serum albumin to check cell viability, which indeed has a long half-life does not fit in a lot. Investigation needs to be done on what actually happens to the liver cells from stem cells to its ultimate form due to their unique ability of regeneration, possibly using the Flow Cytometry techniques (92).

Looking ahead, hepatocyte microencapsulation research should aim to diversify the sources of transplantable cells, minimize the need for immunosuppression, and enhance the survival and functionality of transplanted hepatocytes. Such efforts will not only extend the applicability and safety of hepatocyte microencapsulation techniques but will also provide greater insights into liver cell biology and transplantation methodologies, ultimately benefiting a wider spectrum of patients with liver failure.

## Author contributions

HW: Writing – original draft. FJ: Writing – original draft. PR: Writing – original draft. YY: Writing – review & editing. SS: Writing – review & editing. ZY: Funding acquisition, Writing – review & editing. YW: Conceptualization, Funding acquisition, Project administration, Writing – review & editing. LW: Writing – original draft.

## References

[1] Nguyen MP Jain V Iansante V Mitry RR Filippi C Dhawan A. Clinical application of hepatocyte transplantation: current status, applicability, limitations, and future outlook. Expert Rev Gastroenterol Hepatol. (2020) 14:185–96. doi: 10.1080/17474124.2020.1733975 32098516

[2] Sun Z Yuan X Wu J Wang C Zhang K Zhang L. Hepatocyte transplantation: The progress and the challenges. Hepatol Commun. (2023) 7(10):e0266. doi: 10.1097/HC9.0000000000000266 37695736 PMC10497249

[3] Hansel MC Gramignoli R Skvorak KJ Dorko K Marongiu F Blake W. The history and use of human hepatocytes for the treatment of liver diseases: the first 100 patients. Curr Protoc Toxicol. (2014) 62:14 12 1–23. doi: 10.1002/0471140856.tx1412s62 PMC4343212 25378242

[4] Baldini E Cursio R Sousa De G Margara A Honiger J Saint-Paul M-C. Peritoneal implantation of cryopreserved encapsulated porcine hepatocytes in rats without immunosuppression: viability and function. Transplant Proc. (2008) 40:2049–52. doi: 10.1016/j.transproceed.2008.05.038 18675127

[5] Canaple L Rehor A Hunkeler D. Improving cell encapsulation through size control. J Biomater Sci Polym Ed. (2002) 13:783–96. doi: 10.1163/156856202760197410 12296444

[6] Mao SA Glorioso JM Nyberg SL. Liver regeneration. Transl Res. (2014) 163:352–62. doi: 10.1016/j.trsl.2014.01.005 PMC3976740 24495569

[7] Fitzpatrick E Filippi C Jagadisan B Shivapatham D Anand H Lyne M. Intraperitoneal transplant of Hepatocytes co-Encapsulated with mesenchymal stromal cells in modified alginate microbeads for the treatment of acute Liver failure in Pediatric patients (HELP)-An open-label, single-arm Simon’s two stage phase 1 study protocol. PloS One. (2023) 18:e0288185. doi: 10.1371/journal.pone.0288185 37490429 PMC10368261

[8] Miranda JP Rodrigues A Tostões RM Leite S Zimmerman H Carrondo MJT. Extending hepatocyte functionality for drug-testing applications using high-viscosity alginate-encapsulated three-dimensional cultures in bioreactors. Tissue Eng Part C Methods. (2010) 16:1223–32. doi: 10.1089/ten.tec.2009.0784 20184401

[9] Nino-Vasquez IA Muñiz-Márquez D Ascacio-Valdés JA Contreras-Esquivel JC Aguilar CN Rodríguez-Herrera R. Co-microencapsulation: a promising multi-approach technique for enhancement of functional properties. Bioengineered. (2022) 13:5168–89. doi: 10.1080/21655979.2022.2037363 PMC8973973 35172666

[10] Tran NM Dufresne M Helle F Hoffmann TW François C Brochot E. Alginate hydrogel protects encapsulated hepatic HuH-7 cells against hepatitis C virus and other viral infections. PloS One. (2014) 9:e109969. doi: 10.1371/journal.pone.0109969 25310111 PMC4195705

[11] Lan SF Safiejko-Mroczka B Starly B. Long-term cultivation of HepG2 liver cells encapsulated in alginate hydrogels: a study of cell viability, morphology and drug metabolism. Toxicol In Vitro. (2010) 24:1314–23. doi: 10.1016/j.tiv.2010.02.015 20171269

[12] Azandeh S Nejad DB Bayati V Shakoor F Varaa N Cheraghian B. High mannoronic acid containing alginate affects the differentiation of Wharton’s jelly-derived stem cells to hepatocyte-like cell. J Adv Pharm Technol Res. (2019) 10:9–15. doi: 10.4103/japtr.JAPTR_312_18 30815382 PMC6383346

[13] Jitraruch S Dhawan A Hughes RD Filippi C Soong D Philippeos C. Alginate microencapsulated hepatocytes optimised for transplantation in acute liver failure. PloS One. (2014) 9:e113609. doi: 10.1371/journal.pone.0113609 25438038 PMC4249959

[14] Pasqua M Pereira U Messina A Lartigue C Vigneron P Dubart-Kupperschmitt A. HepaRG self-assembled spheroids in alginate beads meet the clinical needs for bioartificial liver. Tissue Eng Part A. (2020) 26:613–22. doi: 10.1089/ten.tea.2019.0262 31914890

[15] Durkut S Elcin AE Elcin YM. In vitro evaluation of encapsulated primary rat hepatocytes pre- and post-cryopreservation at -80 degrees C and in liquid nitrogen. Artif Cells Nanomed Biotechnol. (2015) 43:50–61. doi: 10.3109/21691401.2013.837476 24059456

[16] Capone SH Dufresne M Rechel M Fleury M-J Salsac A-V Paullier P. Impact of alginate composition: from bead mechanical properties to encapsulated HepG2/C3A cell activities for in vivo implantation. PloS One. (2013) 8:e62032. doi: 10.1371/journal.pone.0062032 23637958 PMC3636232

[17] Nayak S Dey S Kundu SC. Silk sericin-alginate-chitosan microcapsules: hepatocytes encapsulation for enhanced cellular functions. Int J Biol Macromol. (2014) 65:258–66. doi: 10.1016/j.ijbiomac.2014.01.042 24486492

[18] Tian M Han B Tan H You C. Preparation and characterization of galactosylated alginate-chitosan oligomer microcapsule for hepatocytes microencapsulation. Carbohydr Polym. (2014) 112:502–11. doi: 10.1016/j.carbpol.2014.06.025 25129774

[19] He Y Liu C Xia X Liu L. Conformal microcapsules encapsulating microcarrier-L02 cell complexes for treatment of acetaminophen-induced liver injury in rats. J Mater Chem B. (2017) 5:1962–70. doi: 10.1039/C6TB03033E 32263950

[20] Siltanen C Diakatou M Lowen J Haque A Rahimian A Stybayeva G. One step fabrication of hydrogel microcapsules with hollow core for assembly and cultivation of hepatocyte spheroids. Acta Biomater. (2017) 50:428–36. doi: 10.1016/j.actbio.2017.01.010 PMC5809154 28069506

[21] Khodabakhsh Aghdam S Khoshfetrat AB Rahbarghazi R Jafarizadeh-Malmiri H Khaksar M. Collagen modulates functional activity of hepatic cells inside alginate-galactosylated chitosan hydrogel microcapsules. Int J Biol Macromol. (2020) 156:1270–8. doi: 10.1016/j.ijbiomac.2019.11.164 31760032

[22] Zhang Y Lu J Li Z Zhu D Yu X Li L. Enhanced cellular functions of hepatocytes in the hyaluronate-alginate-chitosan microcapsules. Int J Artif Organs. (2021) 44:340–9. doi: 10.1177/0391398820959345 32969286

[23] Yamada K Aoki T Enami Y Tashiro Y Zehaou Z Koizumi T. An improved encapsulation method for cryopreserving hepatocytes for functional transplantation using a thermo-reversible gelation polymer. In Vivo. (2020) 34:2309–16. doi: 10.21873/invivo.12043 PMC7652459 32871755

[24] Lu J Zhang X Li J Yu L Chen E Zhu D. A new fluidized bed bioreactor based on diversion-type microcapsule suspension for bioartificial liver systems. PloS One. (2016) 11:e0147376. doi: 10.1371/journal.pone.0147376 26840840 PMC4739599

[25] Shi XL Zhang Y Gu J-Y Ding Y-T. Coencapsulation of hepatocytes with bone marrow mesenchymal stem cells improves hepatocyte-specific functions. Transplantation. (2009) 88:1178–85. doi: 10.1097/TP.0b013e3181bc288b 19935371

[26] Fitzpatrick E Wu Y Dhadda P Hughes RD Mitry RR Qin H. Coculture with mesenchymal stem cells results in improved viability and function of human hepatocytes. Cell Transplant. (2015) 24:73–83. doi: 10.3727/096368913X674080 24143888

[27] Li CY Stevens KR Schwartz RE Alejandro BS Huang JH Bhatia SN. Micropatterned cell-cell interactions enable functional encapsulation of primary hepatocytes in hydrogel microtissues. Tissue Eng Part A. (2014) 20:2200–12. doi: 10.1089/ten.tea.2013.0667 PMC4137334 24498910

[28] Elisa M Pimenta J Szabó L Noverraz F Passemard S Meier RPH. Beneficial effects of human mesenchymal stromal cells on porcine hepatocyte viability and albumin secretion. J Immunol Res. (2018) 2018:1078547. doi: 10.1155/2018/1078547 29577046 PMC5822000

[29] Chen Y Yu C Lv G Cao H Yang S Zhang Y. Rapid large-scale culturing of microencapsulated hepatocytes: a promising approach for cell-based hepatic support. Transplant Proc. (2014) 46:1649–57. doi: 10.1016/j.transproceed.2014.03.002 24935342

[30] Zheng MH Lin H-L Qiu L-X Cui Y-L Sun Q-F Chen Y-P. Mixed microencapsulation of rat primary hepatocytes and Sertoli cells improves the metabolic function in a D-galactosamine and lipopolysaccharide-induced rat model of acute liver failure. Cytotherapy. (2009) 11:326–9. doi: 10.1080/14653240802582091 19034719

[31] Satta S Shahabipour F Gao W Lentz SR Perlman S Ashammakhi N. Engineering viral genomics and nano-liposomes in microfluidic platforms for patient-specific analysis of SARS-CoV-2 variants. Theranostics. (2022) 12:4779–90. doi: 10.7150/thno.72339 PMC9254234 35832078

[32] Gao Y Xu J Sun B Jiang H-C. Microencapsulated hepatocytes and islets as in vivo bioartificial liver support system. World J Gastroenterol. (2004) 10:2067–71. doi: 10.3748/wjg.v10.i14.2067 PMC4572335 15237436

[33] Kong D. Co-encapsulation of HNF4α overexpressing UMSCs and human primary hepatocytes ameliorates mouse acute liver failure. Stem Cell Res Ther. (2020) 11(1):449. doi: 10.1186/s13287-020-01962-7 33097090 PMC7583302

[34] Liu ZC Chang T I. Biotechnology. Increased viability of transplanted hepatocytes when hepatocytes are co-encapsulated with bone marrow stem cells using a novel method. Artif Cells Blood Substit Immobil Biotechnol. (2002) 30(2):99–112. doi: 10.1081/BIO-120003191 12027231

[35] Nishikawa M Kojima N Komori K Yamamoto T Fujii T Sakai Y. Enhanced maintenance and functions of rat hepatocytes induced by combination of on-site oxygenation and coculture with fibroblasts. J Biotechnol. (2008) 133:253–60. doi: 10.1016/j.jbiotec.2007.08.041 17936393

[36] Liu ZC Chang TMS. Coencapsulation of hepatocytes and bone marrow stem cells: in vitro conversion of ammonia and in vivo lowering of bilirubin in hyperbilirubemia Gunn rats. Int J Artif Organs. (2003) 26(6):491–7. doi: 10.1177/039139880302600607 12894754

[37] Isoda K Kojima M Takeda M Higashiyama S Kawase M Yagi K. Maintenance of hepatocyte functions by coculture with bone marrow stromal cells. J Biosci Bioengineering. (2004) 97:343–6. doi: 10.1263/jbb.97.343 16233641

[38] Goh Takayama AT Okano T. Identification of differentially expressed genes in hepatocyte/endothelial cell co-culture system. J Tissue Eng. (2007) 13:159. doi: 10.1089/ten.2007.13.159 17518589

[39] Valeria I Dhawan A Masmoudi F Lee CA Fernandez-Dacosta R Walker S. A new high throughput screening platform for cell encapsulation in alginate hydrogel shows improved hepatocyte functions by mesenchymal stromal cells co-encapsulation. Front Med. (2018) 5:216. doi: 10.3389/fmed.2018.00216 PMC6095031 30140676

[40] Ding DC Shyu WC Lin SZ. Mesenchymal stem cells. Cell Transplant. (2011) 20:5–14. doi: 10.3727/096368910X 21396235

[41] Kim K Ohashi K Utoh R Kano K Okano T. Preserved liver-specific functions of hepatocytes in 3D co-culture with endothelial cell sheets. Biomaterials. (2012) 33:1406–13. doi: 10.1016/j.biomaterials.2011.10.084 22118777

[42] Chan HF Zhang Y Leong KW. Efficient one-step production of microencapsulated hepatocyte spheroids with enhanced functions. Small. (2016) 12:2720–30. doi: 10.1002/smll.201502932 PMC4982767 27038291

[43] Lee HJ Son MJ Ahn J Oh SJ Lee M Kim A. Elasticity-based development of functionally enhanced multicellular 3D liver encapsulated in hybrid hydrogel. Acta Biomater. (2017) 64:67–79. doi: 10.1016/j.actbio.2017.09.041 28966094

[44] Cui J Wang H Shi Q Sun T Huang Q Fukuda T. Multicellular co-culture in three-dimensional gelatin methacryloyl hydrogels for liver tissue engineering. Molecules. (2019) 24(9):1762. doi: 10.3390/molecules24091762 31067670 PMC6539120

[45] Chang SH Huang HH Kang PL Wu YC Chang M-H Kuo Ming S. In vitro and in vivo study of the application of volvox spheres to co-culture vehicles in liver tissue engineering. Acta Biomater. (2017) 63:261–73. doi: 10.1016/j.actbio.2017.09.028 28941653

[46] Kim M Lee JY Jones CN Revzin A Tae G. Heparin-based hydrogel as a matrix for encapsulation and cultivation of primary hepatocytes. Biomaterials. (2010) 31:3596–603. doi: 10.1016/j.biomaterials.2010.01.068 PMC2837121 20153045

[47] Gevaert E Billiet T Declercq H Dubruel P Cornelissen R. Galactose-functionalized gelatin hydrogels improve the functionality of encapsulated HepG2 cells. Macromol Biosci. (2014) 14:419–27. doi: 10.1002/mabi.201300320 24821670

[48] Lee HJ Ahn J Jung C-R Jeung Y-J Cho H-S Son MJ. Optimization of 3D hydrogel microenvironment for enhanced hepatic functionality of primary human hepatocytes. Biotechnol Bioeng. (2020) 117:1864–76. doi: 10.1002/bit.27328 32162676

[49] Wang Y Liu H Zhang M Wang H Chen W Qin J. One-step synthesis of composite hydrogel capsules to support liver organoid generation from hiPSCs. Biomater Sci. (2020) 8:5476–88. doi: 10.1039/D0BM01085E 32914807

[50] Tirella A Marca La M Brace L-A Mattei G Aylott JW Ahluwalia A. Nano-in-micro self-reporting hydrogel constructs. J BioMed Nanotechnol. (2015) 11:1451–60. doi: 10.1166/jbn.2015.2085 26295145

[51] Khanal S Bhattarai SR Sankar J Bhandari RK Macdonald JM Bhattarai N. Nano-fibre integrated microcapsules: A nano-in-micro platform for 3D cell culture. Sci Rep. (2019) 9:13951. doi: 10.1038/s41598-019-50380-0 31562351 PMC6765003

[52] Zheng Z Wang H Li J Shi Q Cui J Sun T. 3D construction of shape-controllable tissues through self-bonding of multicellular microcapsules. ACS Appl Mater Interfaces. (2019) 11:22950–61. doi: 10.1021/acsami.9b05108 31252493

[53] Yu W Zhang W Chen Y Song X Tong W Mao Z. Cellular uptake of poly(allylamine hydrochloride) microcapsules with different deformability and its influence on cell functions. J Colloid Interface Sci. (2016) 465:149–57. doi: 10.1016/j.jcis.2015.11.065 26674230

[54] Cui J Wang H Zheng Z Shi Q Sun T Huang Q. Fabrication of perfusable 3D hepatic lobule-like constructs through assembly of multiple cell type laden hydrogel microstructures. Biofabrication. (2018) 11:015016. doi: 10.1088/1758-5090/aaf3c9 30523847

[55] Liu Z Takeuchi M Nakajima M Hu C Hasegawa Y Huang Q. Three-dimensional hepatic lobule-like tissue constructs using cell-microcapsule technology. Acta Biomater. (2017) 50:178–87. doi: 10.1016/j.actbio.2016.12.020 27993637

[56] Moriyama K Naito S Wakabayashi R Goto M Kamiya N. Enzymatically prepared redox-responsive hydrogels as potent matrices for hepatocellular carcinoma cell spheroid formation. Biotechnol J. (2016) 11:1452–60. doi: 10.1002/biot.201600087 27617786

[57] Agarwal T Narayan R Maji S Ghosh SK Maiti TK. Decellularized caprine liver extracellular matrix as a 2D substrate coating and 3D hydrogel platform for vascularized liver tissue engineering. J Tissue Eng Regener Med. (2018) 12:e1678–90. doi: 10.1002/term.2594 29052367

[58] Zhang X Lu J He B Tang L Liu X Zhu D. A tryptophan derivative, ITE, enhances liver cell metabolic functions in vitro. Int J Mol Med. (2017) 39:101–12. doi: 10.3892/ijmm.2016.2825 PMC5179183 27959388

[59] Ravichandran A Murekatete B Moedder D Meinert C Bray LJ. Photocrosslinkable liver extracellular matrix hydrogels for the generation of 3D liver microenvironment models. Sci Rep. (2021) 11:15566. doi: 10.1038/s41598-021-94990-z 34330947 PMC8324893

[60] Zhang J Hu X Zheng G Yao H Liang H. In vitro and in vivo antitumor effects of lupeol-loaded galactosylated liposomes. Drug Delivery. (2021) 28:709–18. doi: 10.1080/10717544.2021.1905749 PMC8032341 33825591

[61] Leroux G Neumann M Meunier CF Fattaccioli A Michiels C Arnould T. Hybrid alginate@TiO2 porous microcapsules as a reservoir of animal cells for cell therapy. ACS Appl Mater Interfaces. (2018) 10:37865–77. doi: 10.1021/acsami.8b15483 30360050

[62] Sk MM Das P Panwar A Tan LP. Synthesis and characterization of site selective photo-crosslinkable glycidyl methacrylate functionalized gelatin-based 3D hydrogel scaffold for liver tissue engineering. Mater Sci Eng C Mater Biol Appl. (2021) 123:111694. doi: 10.1016/j.msec.2020.111694 33812568

[63] Tostoes RM Leite SB Miranda JP Sousa M Wang DIC Carrondo MJT. Perfusion of 3D encapsulated hepatocytes–a synergistic effect enhancing long-term functionality in bioreactors. Biotechnol Bioeng. (2011) 108:41–9. doi: 10.1002/bit.22920 20812261

[64] Rebelo SP Costa R Estrada M Shevchenko V Brito C Alves PM. HepaRG microencapsulated spheroids in DMSO-free culture: novel culturing approaches for enhanced xenobiotic and biosynthetic metabolism. Arch Toxicol. (2015) 89:1347–58. doi: 10.1007/s00204-014-1320-9 25107451

[65] Maguire T Novik E Schloss R Yarmush M. Alginate-PLL microencapsulation: effect on the differentiation of embryonic stem cells into hepatocytes. Biotechnol Bioeng. (2006) 93:581–91. doi: 10.1002/bit.20748 16345081

[66] MacPherson D Bram Y Park J Schwartz RE. Peptide-based scaffolds for the culture and maintenance of primary human hepatocytes. Sci Rep. (2021) 11:6772. doi: 10.1038/s41598-021-86016-5 33762604 PMC7990934

[67] Chen L Zhang Y Li S Wang X Li N Wang Y. Effect of plasma components on the stability and permeability of microcapsule. J BioMed Mater Res A. (2014) 102:2408–16. doi: 10.1002/jbm.a.34907 23946210

[68] Liu T Yi S Liu G Hao X Du T Chen J. Aqueous two-phase emulsions-templated tailorable porous alginate beads for 3D cell culture. Carbohydr Polym. (2021) 258:117702. doi: 10.1016/j.carbpol.2021.117702 33593573

[69] Nagata S Ozawa F Nie M Takeuchi S. 3D culture of functional human iPSC-derived hepatocytes using a core-shell microfiber. PloS One. (2020) 15:e0234441. doi: 10.1371/journal.pone.0234441 32525941 PMC7289419

[70] Mai G Nguyen TH Morel P Mei J Andres A Bosco D. Treatment of fulminant liver failure by transplantation of microencapsulated primary or immortalized xenogeneic hepatocytes. Xenotransplantation. (2005) 12:457–64. doi: 10.1111/j.1399-3089.2005.00248.x 16202069

[71] Hang H Shi X Gu G Wu Y Gu J Ding Y. In vitro analysis of cryopreserved alginate-poly-L-lysine-alginate-microencapsulated human hepatocytes. Liver Int. (2010) 30:611–22. doi: 10.1111/liv.2010.30.issue-4 20070514

[72] Kilbride P Mahbubani KT Saeb-Parsy K Morris GJ. Engaging cold to upregulate cell proliferation in alginate-encapsulated liver spheroids. Tissue Eng Part C Methods. (2017) 23:455–64. doi: 10.1089/ten.tec.2017.0131 28727981

[73] Koizumi T Aoki T Kobayashi Y Yasuda D Izumida Y Jin Z. Long-term maintenance of the drug transport activity in cryopreservation of microencapsulated rat hepatocytes. Cell Transplant. (2007) 16:67–73. doi: 10.3727/000000007783464489 17436856

[74] Jitraruch S Dhawan A Hughes RD Filippi C Lehec SC Glover L. Cryopreservation of hepatocyte microbeads for clinical transplantation. Cell Transplant. (2017) 26:1341–54. doi: 10.1177/0963689717720050 PMC5680969 28901189

[75] Lee JH Lee DH Park JK Kim SK Kwon CHD Lee SK. Effect of fulminant hepatic failure porcine plasma supplemented with essential components on encapsulated rat hepatocyte spheroids. Transplant Proc. (2012) 44:1009–11. doi: 10.1016/j.transproceed.2012.01.106 22564611

[76] Rahman TM Diakanov I Selden C Hodgson H. Co-transplantation of encapsulated HepG2 and rat Sertoli cells improves outcome in a thioacetamide induced rat model of acute hepatic failure. Transpl Int. (2010) 18(8):1001–9. doi: 10.1111/j.1432-2277.2005.00156.x 16008752

[77] Yang G Mahadik B Mollot T Pinsky J Jones A Robinson A. Engineered liver tissue culture in an in vitro tubular perfusion system. Tissue Eng Part A. (2020) 26:1369–77. doi: 10.1089/ten.tea.2020.0213 PMC7759278 33054685

[78] Kukla DA Crampton AL Wood DK Khetani SR. Microscale collagen and fibroblast interactions enhance primary human hepatocyte functions in three-dimensional models. Gene Expr. (2020) 20:1–18. doi: 10.3727/105221620X15868728381608 32290899 PMC7284102

[79] Zhang Y Chen XM Sun DL. Effects of coencapsulation of hepatocytes with adipose-derived stem cells in the treatment of rats with acute-on-chronic liver failure. Int J Artif Organs. (2014) 37:133–41. doi: 10.5301/ijao.5000284 PMC6161594 24619896

[80] Teng Y Wang Y Li S Wang W Gu R Guo X. Treatment of acute hepatic failure in mice by transplantation of mixed microencapsulation of rat hepatocytes and transgenic human fetal liver stromal cells. Tissue Eng Part C Methods. (2010) 16(5):1125–34. doi: 10.1089/ten.tec.2009.0374 20121581

[81] Liyuan Wang J Wen X Wang H Wang Y Lin Q. Transplantation of co-microencapsulated hepatocytes and HUVECs for treatment of fulminant hepatic failure. Int J Artif Organs. (2012) 35(6):458–65. doi: 10.5301/ijao.5000092 22562371

[82] Liu WM Zhou X Chen C-Y Lv D-D Huang W-J Peng Y. Establishment of functional liver spheroids from human hepatocyte-derived liver progenitor-like cells for cell therapy. Front Bioeng Biotechnol. (2021) 9:738081. doi: 10.3389/fbioe.2021.738081 34858956 PMC8630579

[83] Song W Lu Y-C Frankel AS An D Schwartz RE Ma M. Engraftment of human induced pluripotent stem cell-derived hepatocytes in immunocompetent mice via 3D co-aggregation and encapsulation. Sci Rep. (2015) 5:16884. doi: 10.1038/srep16884 26592180 PMC4655358

[84] Yuan X Wu J Sun Z Cen J Shu Y Wang C. Preclinical efficacy and safety of encapsulated proliferating human hepatocyte organoids in treating liver failure. Cell Stem Cell. (2024) 31(4):484–98.e5. doi: 10.1016/j.stem.2024.02.005 38458193

[85] Mei J Mai G Baertschiger R Gonelle-Gispert C Serre-Beinier V. Improved survival of fulminant liver failure by transplantation of microencapsulated cryopreserved porcine hepatocytes in mice. Cell Transplant. (2009) 18:101–10. doi: 10.3727/096368909788237168 19476213

[86] Sgroi A Mai G Morel P Baertschiger RM Gonelle-Gispert C Serre-Beinier V. Transplantation of Encapsulated Hepatocytes during Acute Liver Failure Improves Survival without Stimulating Native Liver Regeneration. Cell Transplant. (2011) 20:1791–803. doi: 10.3727/096368911X564976 21396154

[87] Meier RPH Navarro-Alvarez N Morel P Schuurman H-J Strom S Bühler LH. Current status of hepatocyte xenotransplantation. Int J Surg. (2015) 23:273–9. doi: 10.1016/j.ijsu.2015.08.077 26361861

[88] Ham DS Song M-S Park H-S Rhee M Yang HK Lee S-H. Successful xenotransplantation with re-aggregated and encapsulated neonatal pig liver cells for treatment of mice with acute liver failure. Xenotransplantation. (2015) 22:249–59. doi: 10.1111/xen.12177 26174875

[89] Machaidze Z Yeh H Wei L Schuetz C Carvello M Sgroi A. Testing of microencapsulated porcine hepatocytes in a new model of fulminant liver failure in baboons. Xenotransplantation. (2017) 24(3). doi: 10.1111/xen.12297 28261903

[90] Varaa N Azandeh S Khorsandi L Nejad DB Bayati V Bahreini A. Ameliorating effect of encapsulated hepatocyte-like cells derived from umbilical cord in high mannuronic alginate scaffolds on acute liver failure in rats. Iran J Basic Med Sci. (2018) 21:928–35. doi: 10.22038/IJBMS.2018.27928.6847 PMC6272072 30524693

[91] Bonavita AG Quaresma K Cotta-de-Almeida V Pinto MA Saraiva RM Alves LA. Hepatocyte xenotransplantation for treating liver disease. Xenotransplantation. (2010) 17:181–7. doi: 10.1111/xen.2010.17.issue-3 20636538

[92] Gu Y Zheng X Ji J. Liver cancer stem cells as a hierarchical society: yes or no? Acta Biochim Biophys Sin (Shanghai). (2020) 52:723–35. doi: 10.1093/abbs/gmaa050 32490517

